# Response and progression-free survival according to planned treatment duration in patients with relapsed multiple myeloma treated with carfilzomib, lenalidomide, and dexamethasone (KRd) versus lenalidomide and dexamethasone (Rd) in the phase III ASPIRE study

**DOI:** 10.1186/s13045-018-0583-7

**Published:** 2018-04-04

**Authors:** Meletios Dimopoulos, Michael Wang, Vladimir Maisnar, Jiri Minarik, William Bensinger, Maria-Victoria Mateos, Mihaela Obreja, Julie Blaedel, Philippe Moreau

**Affiliations:** 1 School of Medicine, National and Kapodistrian University of Athens, Athens, Greece; 2 The University of Texas MD Anderson Cancer Center, Houston, TX USA; 3 Faculty Hospital and Medical Faculty of Charles University in Hradec Kralove, Hradec Kralove, Czech Republic; 4 University Hospital Olomouc, Medical Faculty of Palacky University Olomouc, Olomouc, Czech Republic; 5 Fred Hutchinson Cancer Research Center, Seattle, WA USA; 6 Hospital Clinico Universitario de Salamanca-IBSAL, Salamanca, Spain; 7 Amgen, Inc., Thousand Oaks, CA USA; 8 University of Nantes, Nantes, France

**Keywords:** Clinical research, Clinical trials, Multiple myeloma, Myeloma therapy

## Abstract

**Background:**

In ASPIRE, carfilzomib, lenalidomide, and dexamethasone (KRd) significantly improved progression-free survival (PFS) and response rates versus lenalidomide and dexamethasone (Rd) in patients with relapsed multiple myeloma. Per protocol, patients received KRd for a maximum of 18 cycles followed by Rd to progression, so the benefit/risk profile of KRd to progression was not established.

**Methods:**

This post hoc analysis evaluated the efficacy and safety of KRd versus Rd at 18 months from randomization. Cumulative rates of complete response (CR) or better over time and PFS hazard ratio (HR) at 18 months were evaluated for KRd versus Rd. PFS HRs were also assessed according to cytogenetic risk, prior lines of therapy, and prior bortezomib treatment. Cox regression analysis was used to evaluate PFS HRs.

**Results:**

The hazard ratio (HR) for PFS at 18 months was 0.58 versus 0.69 for the overall ASPIRE study. Patients with high-risk cytogenetics, ≥ 1 prior lines of therapy, and prior bortezomib exposure benefited from KRd up to 18 months versus Rd. The HRs for PFS at 18 months in the pre-defined subgroups were lower than those in the overall study. The difference in the proportion of KRd and Rd patients achieving at least a complete response (CR) increased dramatically over the first 18 months and then remained relatively constant. The safety profile at 18 months was consistent with previous findings.

**Conclusions:**

The improved PFS HR at 18 months and the continued increase in CR rates for KRd through 18 cycles suggest that there may be a benefit of continued carfilzomib treatment.

**Trial registration:**

Clinical trials.gov NCT01080391. Registered 2 March 2010.

## Background

Multiple myeloma (MM) is a hematologic malignancy characterized by clonal proliferation of plasma cells in the bone marrow [1]. According to Globocan cancer incidence and mortality statistics, the estimated incidence of MM worldwide in 2012 was 114,251, and approximately 80,019 deaths attributed to the disease were reported [2]. MM remains an incurable disease characterized by a recurring pattern of relapse and remission. Upon relapse, patients face poor outcomes, which worsen with each relapse as a response to treatment, duration of response, and health-related quality of life decline [3–7]. In addition, the cost of managing the disease increases [8–10]. Recent advances in the treatment of MM have led to improvements in depth of response, progression-free survival (PFS), and overall survival (OS) in patients with MM [11–13]. Despite improvements in clinical outcome, almost all patients with MM eventually relapse. With many treatment options available for MM patients, there is a need to understand the optimal use of these therapies to achieve an appropriate balance between efficacy and safety. Based on positive clinical study results showing improvements in PFS and OS, there has been a shift to administer continuous therapy for both transplant-eligible and transplant-ineligible MM patients in place of a fixed-dose treatment [14–20]. The rationale for continuous therapy is to control minimal residual disease in order to delay disease recurrence [21].

Carfilzomib is a potent, irreversible, and selective proteasome inhibitor that has shown robust activity in myeloma, both as a single agent and in combination with other anti-myeloma agents [22–26]. Of note, carfilzomib has shown favorable clinical outcomes in patients with relapsed and/or refractory MM with a lower incidence of peripheral neuropathy when compared head-to-head with bortezomib [22]. The dosing and administration schedule of carfilzomib have evolved as the drug has progressed through clinical studies. In the phase Ib/II study (PX-171-006) of carfilzomib in combination with lenalidomide and dexamethasone (KRd), carfilzomib was administered as a 10-min intravenous (IV) infusion initiated at 15 mg/m^2^ and escalated to a maximal dose of 27 mg/m^2^ in patients with relapsed MM [27]. The overall response rate was 77% in patients who received the maximum planned dose of carfilzomib (27 mg/m^2^), and adverse events were consistent with the known safety profiles of the three agents [27, 28]. Based on these findings, the carfilzomib dose of 27 mg/m^2^ was selected for further evaluation in the ASPIRE study [25].

The ASPIRE study evaluated KRd in comparison with lenalidomide and dexamethasone (Rd) alone in patients with relapsed MM who had received one to three prior lines of therapy. The addition of carfilzomib to Rd resulted in significantly higher overall response rate versus Rd alone (87.1 vs 66.7%; *p* < 0.001) and longer PFS (26.3 vs Rd, 17.6 months; hazard ratio [HR] 0.69; 95% confidence interval [CI] 0.57–0.83) [25]. In addition, results from the final OS analysis of ASPIRE showed an almost 8-month improvement in OS in patients treated with KRd compared with Rd (48.3 vs 40.4 months; HR 0.79; 95% CI 0.67–0.95; *p* = 0.0045) [29]. Carfilzomib treatment was discontinued after 18 cycles because data on the long-term safety of carfilzomib were not yet available when the ASPIRE study was initiated. Because carfilzomib was discontinued after 18 cycles, the optimal duration of KRd treatment was not determined. Herein, we performed a post hoc analysis of the ASPIRE study to compare the safety and efficacy of KRd versus Rd at 18 months from randomization (i.e., at the latest time that allowed a direct comparison of KRd vs Rd).

## Methods

The ASPIRE study has been previously described in detail by Stewart et al. [25]. The primary endpoint of the study was PFS. Secondary endpoints included OS, overall response rate (partial response or better), and safety. Prior bortezomib therapy was allowed, provided that patients did not have disease progression during treatment. Prior Rd treatment was also permitted if patients had not progressed during the first 3 months of therapy or discontinued treatment due to intolerance. All patients were required to have adequate renal, hematological, and hepatic function at screening. Patients who had grade 3 or 4 peripheral neuropathy (or grade 2 with pain) within 14 days before randomization or New York Heart Association class III or IV heart failure were excluded. All patients provided informed consent, and the study was performed according to the Declaration of Helsinki. The study protocol was approved by the institutional review boards of all participating institutions.

Patients were randomized 1:1 to receive KRd or Rd in 28-day cycles until the withdrawal of consent, disease progression, or the occurrence of toxicity. The stratification factors used for randomization were prior bortezomib therapy, prior lenalidomide therapy, and β2-microglobulin levels. Patients were administered carfilzomib on days 1, 2, 8, 9, 15, and 16 of cycles 1–12 and on days 1, 2, 15, and 16 of cycles 13–18. Carfilzomib was given as a 10-min infusion at a starting dose of 20 mg/m^2^ on days 1 and 2 of cycle 1, and 27 mg/m^2^ thereafter. In the KRd and Rd arms, lenalidomide (25 mg) was given on days 1–21, and dexamethasone (40 mg) was given on days 1, 8, 15, and 22. Carfilzomib was discontinued after cycle 18 after which all patients on the carfilzomib arm continued to receive Rd treatment until disease progression.

Cumulative rates of complete response or better (≥ CR) over time were evaluated from the start of the treatment for KRd versus Rd. The PFS HR at 18 months for the intent-to-treat population was evaluated using the piecewise Cox regression analysis [30]. An analysis of PFS within the carfilzomib arm among patients who achieved ≥ CR compared with patients who achieved < CR was performed using the Simon-Makuch landmark transient state approach [31]. Patients were classified as ≥ CR or < CR according to their best overall response (BOR) at the landmark time. The choice of landmark time was based on the median time to reach a BOR of ≥ CR or better, which was 6.7 months for the KRd arm and 8.3 months for the Rd arm. PFS events noted by landmark time were excluded from the analysis. This landmark method was used to minimize the bias in favor of responders represented by the time required to reach the response.

PFS HRs were also evaluated in subgroups of patients in ASPIRE according to cytogenetic risk, prior lines of therapy and prior bortezomib treatment. All patients who received at least one dose of study treatment were evaluated for safety analyses. The International Myeloma Working Group Uniform Response Criteria were used to assess response and disease progression [32]. According to these criteria, ≥ CR was defined as CR (i.e., negative immunofixation on the serum and urine, disappearance of any soft tissue plasmacytomas, and < 5% plasma cells in bone marrow) plus stringent CR (i.e., normal free light chain ratio and absence of clonal cells in bone marrow by immunohistochemistry or immunofluorescence) [32].

## Results

The cutoff date for the primary PFS analysis in ASPIRE was June 16, 2014, after a median follow-up of over 30 months. A total of 792 patients were enrolled in the intent-to-treat population, and 396 each were randomized to the KRd and Rd arms. As reported in the primary ASPIRE publication [25], patient demographic and baseline disease characteristics for the intent-to-treat population were generally balanced between treatment arms, including cytogenetic risk, baseline neuropathy, number of prior regimens, and prior therapies.

**Fig. 1:**
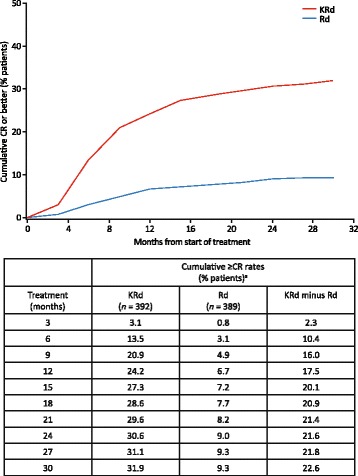
Cumulative ≥CR rates by time. ≥CR, complete response or better; <CR, less than complete response; KRd, carfilzomib, lenalidomide, and dexamethasone; Rd, lenalidomide and dexamethasone. ^a^Data are from safety population: cumulative ≥CR rates achieved according to time from treatment start

The overall PFS HR in ASPIRE for KRd versus Rd was 0.69 (95% CI 0.57–0.83). At 18 months from randomization, the PFS HR was 0.58 (95% CI 0.46–0.72) in favor of the KRd group (Table 1). PFS HRs at 18 months also favored KRd versus Rd in patients with high-risk cytogenetics (HR 0.56; 95% CI 0.31–0.99), standard cytogenetic risk (HR 0.54; 95% CI 0.37–0.80), one prior line of treatment (HR 0.58; 95% CI 0.41–0.82), two or more prior lines of treatment (HR 0.60; 95% CI 0.45–0.81), and prior bortezomib exposure (HR 0.59; 95% CI 0.45–0.78) (Table 1). HRs were numerically lower at 18 months from randomization compared with HRs for the same subgroups from the primary ASPIRE study [25]. In the overall ASPIRE study, a total of 126 (32%) patients in the KRd arm and 37 (9%) in the Rd arm achieved ≥ CR, with a median time from treatment start to ≥ CR of 6.7 months for KRd and 8.3 months for Rd. The cumulative ≥ CR rates are presented in Fig. 1. Cumulative ≥ CR rates increased for both KRd-treated and Rd-treated patients over the course of 30 months, but ≥ CR rates were higher for KRd versus Rd across all time points. At 18 months from randomization, 28.6% of patients who received KRd versus 7.7% of patients who received Rd achieved ≥ CR (Fig. 1). There was a marked separation between the ≥ CR curves of KRd-treated and Rd-treated patients in the first 9 to 12 months of treatment with ≥ CR rates rising rapidly in the KRd arm. The ≥ CR curves of KRd- and Rd-treated patients continued to separate from 12 to 18 months and then remained approximately parallel to each other.

**Table 1:** PFS HRs for the ASPIRE ITT population and select subgroups

	At 18 months from randomization	Overall ASPIRE study [25, 36, 46]
	HR (KRd/Rd) (95% CI)	
Entire ASPIRE population	0.58 (0.46–0.72)	0.69 (0.57–0.83)
Cytogenetic risk		
High^a^	0.56 (0.31–0.99)	0.70 (0.43–1.16)
Standard	0.54 (0.37–0.80)	0.66 (0.48–0.90)
Prior lines of treatment		
1	0.58 (0.41–0.82)	0.71 (0.53–0.96)
2	0.65 (0.44–0.97)	0.75 (0.54–1.04)
3	0.53 (0.34–0.84)	0.68 (0.47–1.00)
Prior bortezomib treatment	0.59 (0.45–0.78)	0.70 (0.56–0.88)
No prior bortezomib treatment	0.58 (0.39–0.86)	0.73 (0.52–1.02)

*CI* confidence interval, *HR* hazard ratio, *ITT* intent-to-treat, *KRd* carfilzomib, lenalidomide, and dexamethasone, *PFS* progression-free survival, *Rd* lenalidomide and dexamethasone

^a^High cytogenetic risk was defined by t(4;14), t(14;16), or del(17p) in ≥ 60% of plasma cells. At baseline, 12.1% of KRd-treated patients and 13.1% of Rd-treated patients had high-risk cytogenetics

**Fig. 2:**
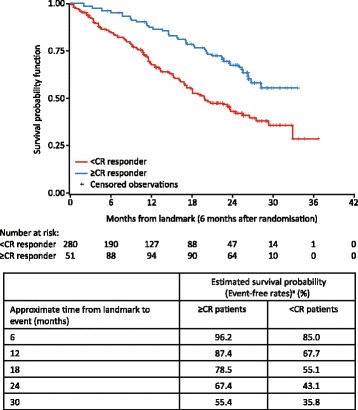
PFS in KRd patients: ≥CR responders versus <CR responders in overall ASPIRE population. ≥CR, complete response or better; <CR, less than complete response; KRd, carfilzomib, lenalidomide and dexamethasone; PFS, progression-free survival. Based on Simon-Makuch Landmark analysis at 6 months after randomization (intent-to-treat population)

**Fig. 3:**
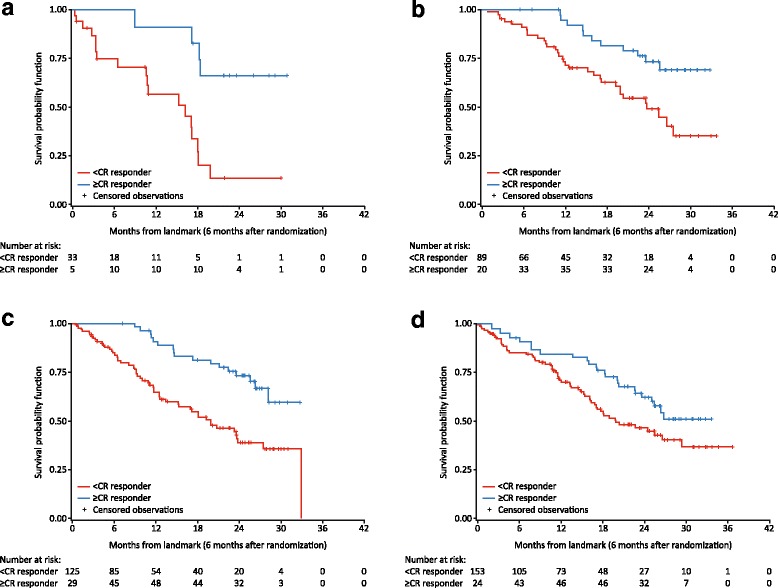
PFS in KRd patients: ≥CR responders versus <CR responders by select subgroups in ASPIRE. **a** High-risk cytogenetics. **b** Prior bortezomib therapy. **c** One prior line of treatment. **d** Two or more prior lines of treatment. ≥CR, complete response or better; <CR, less than complete response; KRd, carfilzomib, lenalidomide and dexamethasone; PFS, progression-free survival

PFS rates were compared for KRd-treated patients who achieved ≥ CR with KRd-treated patients who achieved < CR in the overall ASPIRE population after 6 months from randomization (Fig. 2). KRd-treated patients who achieved ≥ CR were associated with higher PFS rates compared with KRd-treated patients who achieved < CR. Specifically, PFS rates for ≥ CR patients were 96, 87, 79, 67, and 55% at approximately 6, 12, 18, 24, and 30 months from the landmark (6 months from randomization), respectively, and PFS rates for < CR patients were 85, 68, 55, 43, and 36% at 6, 12, 18, 24, and 30 months from the landmark, respectively (Fig. 2). In the high-risk cytogenetics, prior bortezomib exposure, and one or more prior lines of treatment subgroups, KRd patients who achieved ≥ CR were also associated with higher PFS rates at all observable time points from the landmark than KRd patients who achieved < CR (Fig. 3).

At 18 months from randomization, median treatment duration was 78 weeks (95% CI 1–80) in the KRd arm and 57 weeks (95% CI 1–79) in the Rd arm, and the median number of cycles for KRd versus Rd was 19 (95% CI 1–20) versus 14 (95% CI 1–21). Disease progression was the most common reason for treatment discontinuation in the first 18 months of treatment, and treatment discontinuations due to disease progression were more frequent with Rd (36.2%) compared with KRd (20.9%). The rates of treatment-emergent adverse events, discontinuation, and deaths at 18 months from randomization are presented in Table 2. Rates of events (KRd vs Rd) were 96.7 versus 95.6% for any-grade adverse events and 80.4 versus 75.3% for grade ≥ 3 adverse events (Table 2). Serious adverse events occurred in 53.8% of patients treated with KRd and 46.3% of patients treated Rd. A total of 83 (21.2%) patients receiving KRd and 81 (20.8%) patients receiving Rd discontinued any study drug due to adverse events (Table 2). Deaths due to adverse events occurred in 25 (6.4%) KRd-treated patients and 29 (7.5%) Rd-treated patients (Table 2). Rates of any-grade thrombocytopenia (27.3 vs 21.6%) and grade ≥ 3 thrombocytopenia (16.1 vs 12.1%) were higher for KRd versus Rd arm. The frequency of any-grade acute renal failure was similar between the KRd and Rd arms (KRd 6.6%; Rd 6.2%), but grade ≥ 3 acute renal failure occurred slightly more frequently in the Rd arm than the KRd arm (KRd 2.3%; Rd 3.1%). Any-grade and grade ≥ 3 anemia and neutropenia occurred slightly more frequently in the KRd arm than the Rd arm. The incidence of any-grade peripheral neuropathy was 15.1% for KRd and 14.1% for Rd. Grade ≥ 3 peripheral neuropathy occurred at the same rate in the KRd and Rd arms (KRd 2.3%; Rd 2.3%). The incidence of cardiovascular events including any-grade hypertension (13.0 vs 5.9%), cardiac failure (5.9 vs 3.3%), and dyspnea (20.9 vs 17.2%) was higher for KRd versus Rd. Grade ≥ 3 hypertension (4.1 vs 1.3%), cardiac failure (3.6 vs 1.3%), and dyspnea (2.6 vs 1.5%) occurred more frequently in the KRd arm than the Rd arm.

**Table 2:** Treatment-emergent adverse events, discontinuation, and deaths at 18 months from randomization (safety population)

	KRd (*n* = 392)	Rd (*n* = 389)
Patients with any-grade AE, *n* (%)	379 (96.7)	372 (95.6)
Grade ≥ 3 AE, *n* (%)	315 (80.4)	293 (75.3)
Serious AEs, *n* (%)	211 (53.8)	180 (46.3)
AE leading to treatment discontinuation, *n* (%)	83 (21.2)	81 (20.8)
AE leading to death, *n* (%)	25 (6.4)	29 (7.5)
Any-grade AEs of interest, *n* (%)		
Anemia	157 (40.1)	147 (37.8)
Thrombocytopenia	107 (27.3)	84 (21.6)
Neutropenia	137 (34.9)	126 (32.4)
Hypertension	51 (13.0)	23 (5.9)
Dyspnea (HLT)	82 (20.9)	67 (17.2)
Peripheral neuropathy (SMQN)	59 (15.1)	55 (14.1)
Cardiac failure (SMQN)	23 (5.9)	13 (3.3)
Acute renal failure (SMQN)	26 (6.6)	24 (6.2)
Grade ≥ 3 AEs of interest, *n* (%)		
Anemia	69 (17.6)	65 (16.7)
Thrombocytopenia	63 (16.1)	47 (12.1)
Neutropenia	111 (28.3)	99 (25.4)
Hypertension	16 (4.1)	5 (1.3)
Dyspnea	10 (2.6)	6 (1.5)
Peripheral neuropathy (SMQN)	9 (2.3)	9 (2.3)
Cardiac failure (SMQN)	14 (3.6)	5 (1.3)
Acute renal failure (SMQN)	9 (2.3)	12 (3.1)

*AE* adverse event, *HLT* high-level term, *KRd* carfilzomib, lenalidomide, and dexamethasone, *Rd* lenalidomide and dexamethasone, *SMQN* standardized Medical Dictionary for Regulatory Activities query, narrow scope

## Discussion

Previously reported results from the phase III ASPIRE study showed that the addition of carfilzomib to Rd resulted in high ≥ CR rates and a clinically meaningful and statistically significant improvement in PFS and OS in patients with relapsed MM who had received at least one to three prior lines of therapy [25, 29]. Importantly, per protocol, carfilzomib application on days 8 and 9 was omitted after cycle 12, and carfilzomib treatment was completely discontinued after 18 cycles (Rd continued until progression). Notably, the PFS benefit observed in ASPIRE was not a result of poor outcomes in the Rd group as the reported median PFS in the Rd group was similar to that observed in other trials comparing triplet combinations to Rd [33–35]. For the primary PFS analysis, the median follow-up was 32.3 months in the KRd group and 31.5 months in the Rd group. An analysis of ASPIRE by previous treatment showed benefits in PFS with KRd versus Rd in patients with one prior line of therapy, ≥ 2 previous lines of therapy, and previous bortezomib exposure over an approximately 3-year time period, including at 18 months [36].

In this post hoc analysis, we evaluated the efficacy and safety of KRd versus Rd at 18 months from randomization to enable a more robust evaluation of the addition of carfilzomib to Rd. We found that for the first 18 months since randomization, deeper responses were achieved with KRd compared with Rd, and the PFS HR for KRd versus Rd was lower than that for the overall study, indicating a better treatment effect than that reported for the entire duration of the ASPIRE study. Specifically, treatment with KRd for 18 months reduced the risk of progression or death by 42%. The rate of increase in the proportion of patients achieving ≥ CR was higher in the KRd arm compared with the Rd arm, particularly in the first 15 months from the start of treatment. It is known that a positive correlation exists between the depth of response and improved outcomes in terms of PFS and OS in patients with MM [37–39]. We found that the proportion of patients who reached ≥ CR was approximately three to four times higher in the KRd than Rd arm (at 18 months from randomization, KRd 28.6%; Rd 7.7%). We compared PFS in KRd-treated patients who reached ≥ CR with KRd-treated patients who achieved < CR. PFS rates in the KRd arm were higher for patients who reached ≥ CR versus patients who achieved < CR at all observable time points in the overall ASPIRE study. These results show that PFS was improved in KRd-treated patients who achieved a deeper response.

We observed an increase in ≥ CR rates in both KRd and Rd treatment arms, with further increase in ≥ CR rates in the KRd arm beyond month 18 (Fig. 1). Patients with high disease burden may have achieved CR later compared with patients with smaller tumor burdens. In addition, there may have been subjective factors that could explain the delay in achieving deeper responses, including to the time the bone marrow tests were performed to confirm CR. For example, investigators may have delayed their decision to perform bone marrow tests based on their overall evaluation of the patient, or these tests were analyzed by a local lab and had to be re-done by the central lab to be considered for inclusion in the clinical data.

In the relapsed setting, it is a common practice to continue treatment until progression, provided that the treatment is well-tolerated. In ASPIRE, treatment with lenalidomide and dexamethasone was continued until disease progression, whereas carfilzomib treatment was omitted after 12 cycles and discontinued after 18 cycles per protocol. In another randomized phase III studies such as TOURMALINE-MM1, ELOQUENT, and POLLUX, with patient populations similar to the population in ASPIRE, treatment was continued until disease progression [33–35]. Based on our findings in this analysis, it can be speculated that if carfilzomib had been administered beyond 18 cycles, the PFS HR might have been improved further.

With respect to safety, the rates of treatment discontinuations and death due to adverse events were similar between the KRd and Rd arms at 18 months from randomization. Hematological adverse events including any-grade and grade ≥ 3 anemia, thrombocytopenia, and neutropenia occurred more frequently in the KRd versus Rd arm at 18 months from randomization. The rates of any-grade and grade ≥ 3 hypertension, dyspnea, and cardiac failure were also higher in patients receiving KRd than patients receiving Rd. The incidence of any-grade and grade ≥ 3 peripheral neuropathy was similar between the KRd and Rd arms. Overall, the safety findings reported in this post hoc analysis were similar to those reported in the safety analysis of the primary ASPIRE study.

Treating MM can be a challenge, in particular for patients with high-risk cytogenetics. High-risk cytogenetics has been shown to have a negative impact on survival and prognosis [40–42]. In ASPIRE, high risk was defined by t(4;14), t(14;16), or del(17p) in ≥ 60% of plasma cells. Of note, the advantage of KRd was retained across subgroups, including patients with high-risk cytogenetics at study entry. In addition, patients with relapsed and/or refractory disease present a challenge, because the benefit they receive from the therapy usually decreases with subsequent lines of therapy [43]. We found that in patients who had high-risk cytogenetics or one or more prior lines of therapy, PFS HRs favored KRd versus Rd in the first 18 months and were lower than the HR for the overall ASPIRE study [25]. As expected, PFS rates were higher for ≥ CR responders than < CR responders in the KRd arm. Similar efficacy results were observed for the subgroup of patients who had prior bortezomib exposure, which is notable as many patients with relapsed MM will have been exposed to bortezomib [44, 45].

We observed that the PFS HR (0.58) for KRd versus Rd for the first 18 months was lower than the PFS HR for the overall ASPIRE study (0.69). The 18-month PFS HRs for patients with high or standard cytogenetic risk, one or two or more prior lines of treatment and prior, and no prior bortezomib exposure were also lower than those reported in the preplanned subgroup analyses of ASPIRE according to cytogenetic risk [46], prior lines of treatment [36], and prior therapy [36]. The lower HRs observed at 18 months are possibly due to patients receiving carfilzomib for a maximum of 18 months. Had patients received continuous carfilzomib treatment in the ASPIRE study, we could have potentially observed even greater clinical benefit in the KRd arm, with longer PFS and deeper responses. Continuous treatment with KRd might have been even more efficacious in patients with high-risk cytogenetics. In fact, studies show that extended duration of therapy is associated with better clinical outcomes in patients with MM. Specifically, Palumbo et al. observed that newly diagnosed MM patients who received novel agent-based continuous therapy had longer PFS and improved OS than patients who had a fixed duration of therapy [19]. In addition, using data from the bortezomib-melphalan arm of the phase III VISTA study, Mateos et al. found that higher cumulative bortezomib dose, which reflects prolonged treatment duration, is associated with improved OS in newly diagnosed MM patients [47]. With respect to carfilzomib, newly diagnosed MM patients who received continuous treatment with KRd achieved high responses [48–50]. Of note, upon approval of KRd, European Medicines Agency concluded that treatment with KRd could continue beyond 18 cycles based on individual risk-benefit assessment, as the data on the tolerability and toxicity of carfilzomib beyond 18 cycles are limited [51]. Although we are not recommending routine administration of carfilzomib in the KRd regimen beyond 18 cycles, this analysis, despite the limitations of the post hoc design, supports further evaluation of carfilzomib treatment beyond 18 cycles. Additional larger studies are required to evaluate whether prolonged carfilzomib treatment duration leads to further improvements in clinical outcomes without additional adverse events.

## Conclusions

This post hoc analysis of ASPIRE shows that KRd provided greater responses and improvements in PFS at 18 months compared with Rd alone. Cumulative ≥ CR rates increased over time in the KRd arm and were higher than the cumulative ≥ CR rates in the Rd arm, with rates rapidly rising in the first 15 months from the start of treatment. PFS HRs for the first 18 months from randomization were lower for KRd versus Rd than in the overall ASPIRE study and regardless of prior cytogenetic status, number of prior lines of treatment, and prior bortezomib exposure. Compared with the overall results of the ASPIRE study, the markedly lower HR for PFS at 18 months in this analysis suggests a potential additional clinical benefit of continued treatment with carfilzomib beyond 18 months. However, further evaluation is warranted.

## Abbreviations

BOR: Best overall response
CR: Complete response
HR: Hazard ratio
IV: Intravenous
KRd: Carfilzomib, lenalidomide, and dexamethasone
MM: Multiple myeloma
OS: Overall survival
PFS: Progression-free survival
Rd: Lenalidomide and dexamethasone
